# Supplementary material for: Quantitative proteomics identifies the universally conserved ATPase Ola1p as a positive regulator of heat shock response in Saccharomyces cerevisiae
Source: J Biol Chem. 2021 Sep 24;297(5):101050. doi: 10.1016/j.jbc.2021.101050 (PMC8531669; doi:10.1016/j.jbc.2021.101050)
Supplement: Figures S1–S6 and Supplemental Table Legends [file mmc7.pdf]

Supporting Information

Quantitative proteomics identifies the universally conserved ATPase Ola1p as a positive regulator of heat shock response in *Saccharomyces cerevisiae*

Stefan Dannenmaier, Christine Desroches Altamirano, Lisa Schüler, Ying Zhang, Johannes Hummel, Martin Milanov, Silke Oeljeklaus, Hans-Georg Koch, Sabine Rospert, Simon Alberti and Bettina Warscheid

Material included:

Supplemental Figures S1 - S6

Supplemental Table Legends (Tables S1 - S6; xlsx files)

References

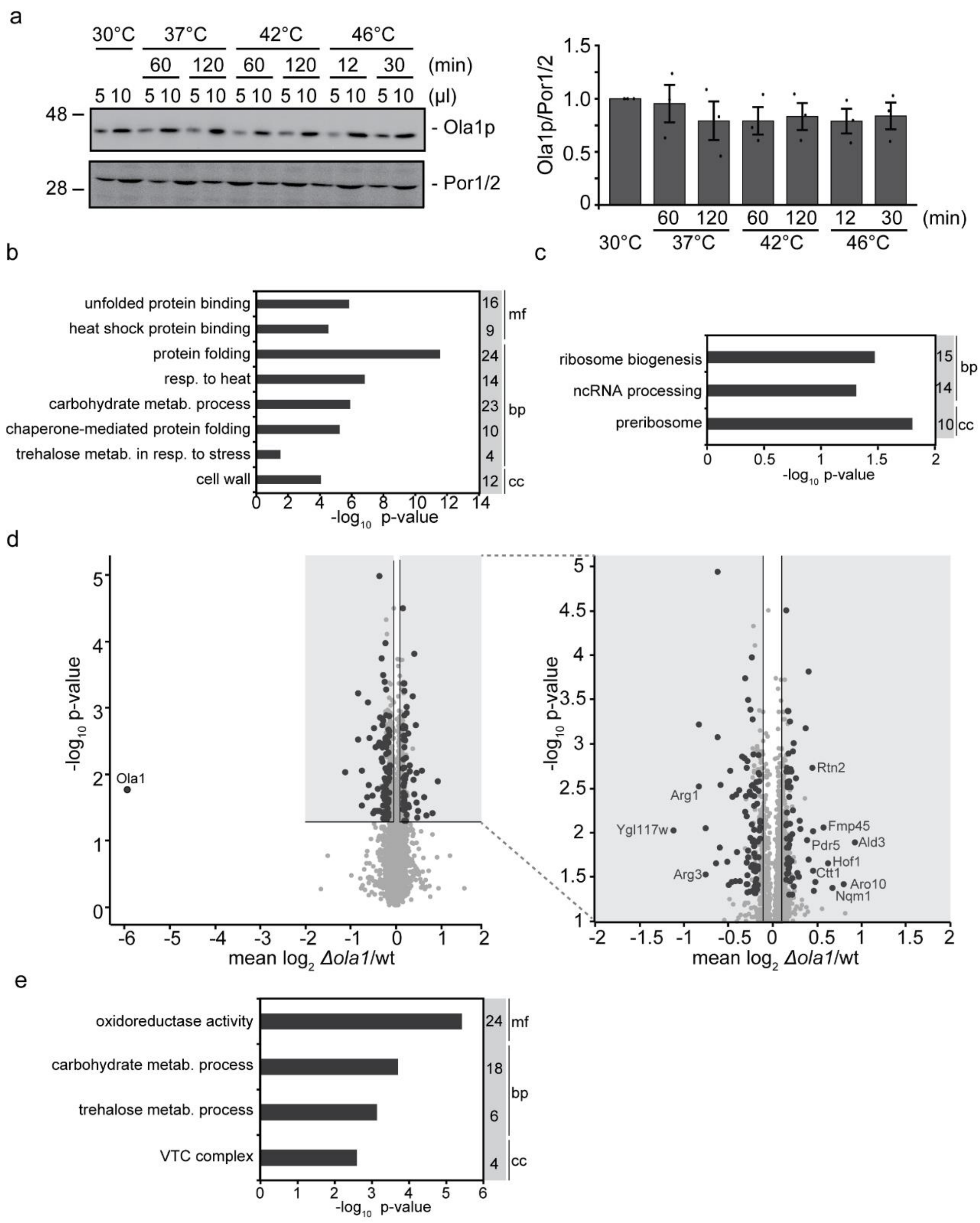

a
30°C 37°C 42°C 46°C
60 120 60 120 12 30 (min)
5 10 5 10 5 10 5 10 5 10 5 10 5 10 (µl)
48
28
- Ola1p
- Por1/2
Ola1p/Por1/2
30°C 37°C 42°C 46°C
b
unfolded protein binding
heat shock protein binding
protein folding
resp. to heat
carbohydrate metab. process
chaperone-mediated protein folding
trehalose metab. in resp. to stress
cell wall
-log10 p-value
mf
bp
cc
c
ribosome biogenesis
ncRNA processing
preribosome
-log10 p-value
d
-log10 p-value
mean log2 Δola1/wt
Ola1
Rtn2
Arg1
Ygl117w
Fmp45
Pdr5
Ald3
Hof1
Ctt1
Arg3
Aro10
Nqm1
e
oxidoreductase activity
carbohydrate metab. process
trehalose metab. process
VTC complex
-log10 p-value

Figure S1

Figure S1. Proteomic effects of heat shock and *OLA1* deletion.

a. Cells were exposed to elevated temperatures as indicated and levels of Ola1p were assessed by immunoblotting (left). Immunoblot signals for Ola1p from three independent biological replicates were quantified and normalized to the loading control Por1/2 (right). Error bars indicate SD.

b, c, e. Gene Ontology term enrichment analysis of proteins significantly increased (b) or decreased (c) in abundance in heat-stressed wildtype cells (related to Figure 1b), and significantly increased in heat-stressed Δ*ola1* cells (e; related to Figure 1d). Numbers indicate the number of proteins assigned to the respective terms for the domains 'cellular component' (cc), 'molecular function' (mf), and 'biological process' (bp). resp., response; metab., metabolism; VTC, vacuolar transporter chaperone.

d. Left, differences in the proteome of wildtype and Δ*ola1* cells under non-stress conditions. Larger circles indicate proteins significantly changed in abundance (i.e., *p*-value < 0.05 for both *t*-test and Significance B; n = 4). Right, Zoom-in of the shaded area of the volcano plot.

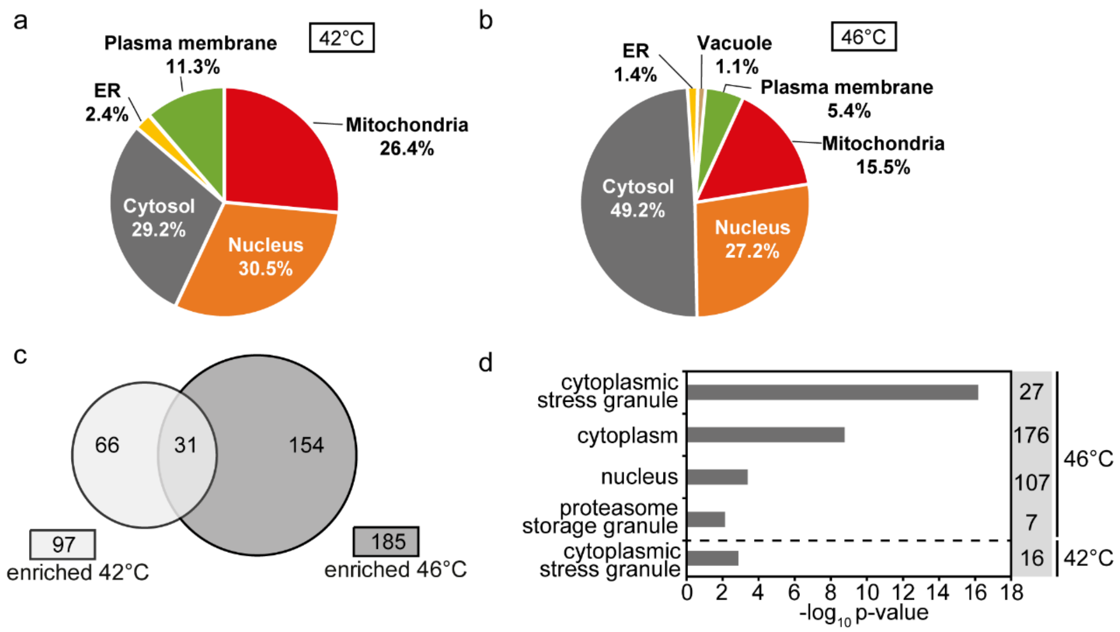

Figure S2

Figure S2: Analysis of detergent-insoluble protein aggregates.

a, b, d. Subcellular localization (a, b) and GO term enrichment analysis (d) of proteins that were at least 2-fold enriched (*p*-value < 0.05) in the NP-40-insoluble pellet fractions of wildtype cells heat-stressed at 42°C for 60 minutes (a) or at 46°C for 12 minutes (b). Numbers in (d) indicate the number of proteins assigned to the respective term for the domain 'cellular component'.

c. Venn diagram illustrating the overlap of proteins identified in the detergent-resistant pellet fraction of wildtype cells heat-stressed at 42°C for 60 minutes or at 46°C for 12 minutes.

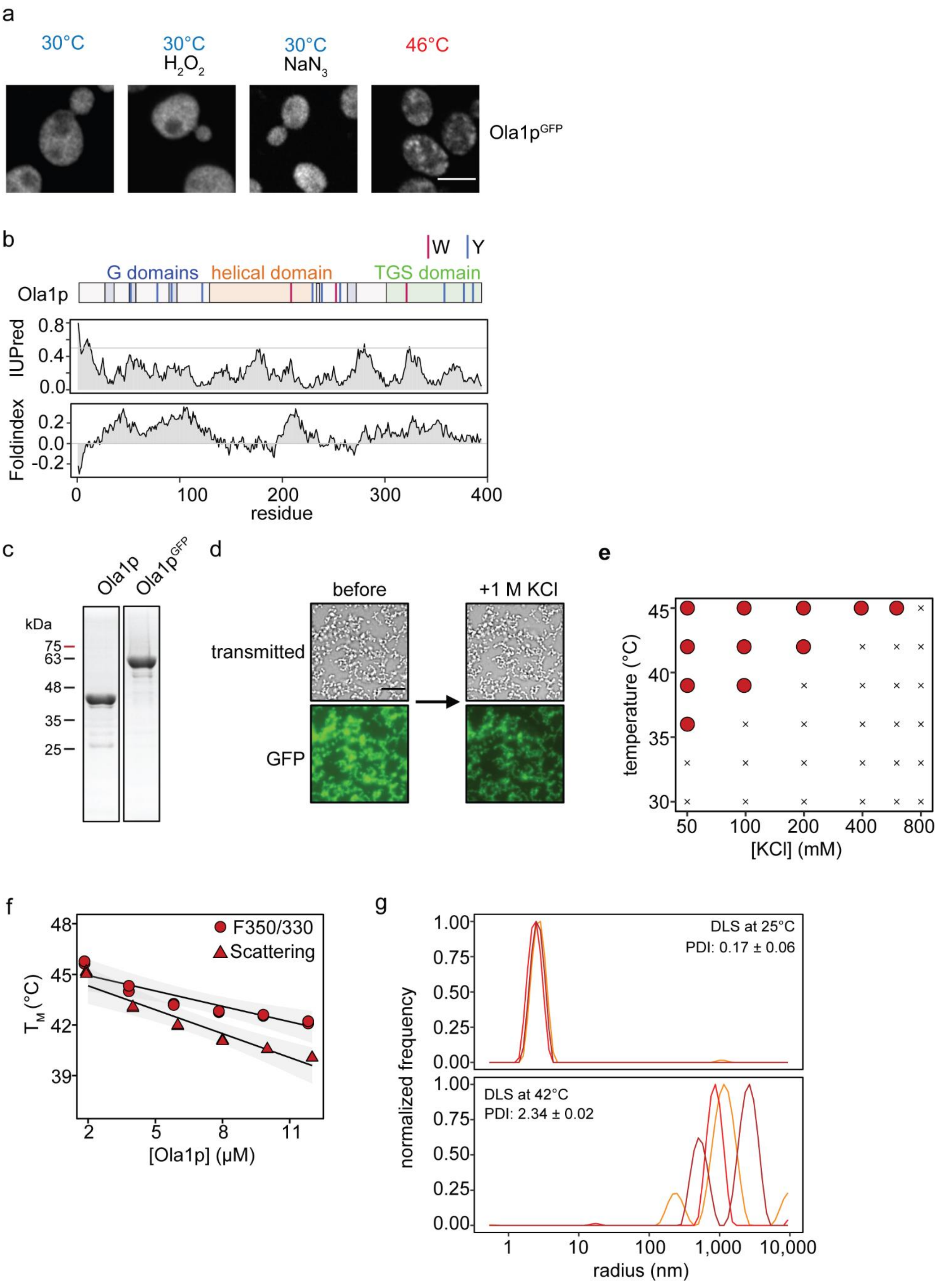
a
30°C
30°C
H2O2
30°C
NaN3
46°C
Ola1pGFP
b
W
Y
G domains
helical domain
TGS domain
Ola1p
IUPred
Foldindex
residue
c
Ola1p
Ola1pGFP
kDa
75
63
48
35
25
d
before
+1 M KCl
transmitted
GFP
e
temperature (°C)
[KCl] (mM)
f
F350/330
Scattering
TM (°C)
[Ola1p] (µM)
g
DLS at 25°C
PDI: 0.17 ± 0.06
DLS at 42°C
PDI: 2.34 ± 0.02
normalized frequency
radius (nm)

Figure S3

Figure S3. Assembly of Ola1p *in vivo* and *in vitro*.

a. Subcellular localization of C-terminally tagged Ola1p^{GFP} in cells exposed to different stress conditions, analyzed by fluorescence microscopy. Stress conditions were as follows: H_2O_2 (1 mM, 30 min), NaN_3 (0.5%, 30 min), and heat stress (46°C, 12 min). Scale bar, 5 µm.

b. Schematic representation of the Ola1p domain structure. Tryptophan (W) and tyrosine (Y) residues are highlighted (top). Shown below are prediction plots depicting intrinsic disorder and folding of Ola1p, generated using the IUPred (1) and Foldindex (2) algorithm, respectively.

c. Coomassie-stained SDS gels of purified Ola1p and Ola1p^{GFP}.

d. Transmitted light and fluorescent microscopy images (GFP) of 2.5 µM Ola1p heat-induced assemblies (1:10 ratio of Ola1p^{GFP} to Ola1p) in pH 7.0 buffer before and after the addition of 1 M KCl. Note that the GFP signal after addition of 1 M KCl decreases due to the dilution of the Ola1p^{GFP} signal. Scale bar, 10 µm.

e. Diagram depicting the temperature and salt concentrations at which Ola1p assembles, as determined by fluorescence microscopy. Red dots represent conditions at which more than 5% of the Ola1p^{GFP} signal was assembled.

f. The T_M values of F350/330 and scattering of Ola1p are plotted as function of Ola1p concentration. Three replicates and a trend line are plotted.

g. Normalized frequency of the distribution of radius measurements for Ola1p at 25°C (top) and 42°C (bottom), as determined by dynamic light scattering (DLS). The distribution of three replicates is plotted and the averaged polydispersity index (PDI) values are indicated.

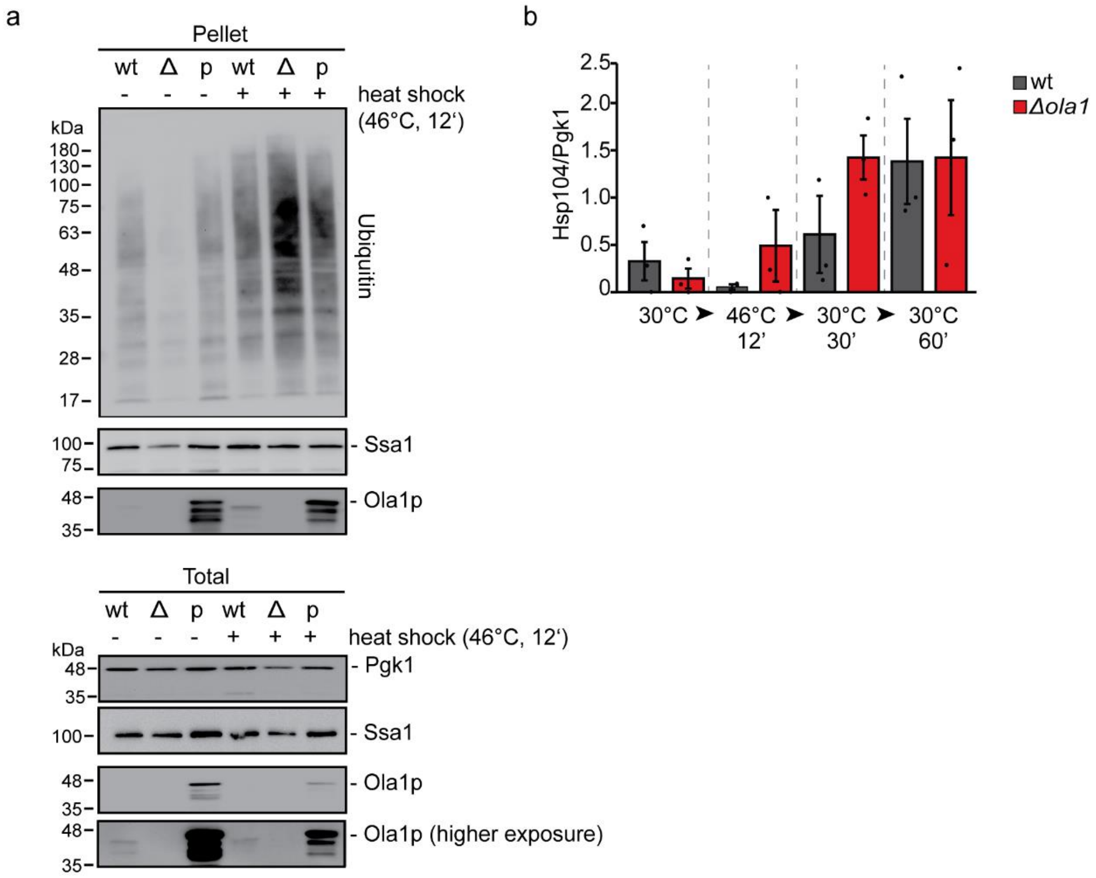

Figure S4

Figure S4: Effects of *OLA1* deletion and overexpression on detergent-resistant protein aggregates.

a. Whole cell extracts (Total) and NP40-insoluble protein aggregates (Pellet) were prepared from wildtype (wt) and *Δola1* cells (Δ) as well as from cells overexpressing plasmid-encoded Ola1p (p). Fractions obtained from heat-stressed (+) and control cells (-) were analyzed by immunoblotting using antibodies directed against the indicated proteins.

b. Levels of Hsp104 in protein aggregates, determined by immunoblotting as exemplarily shown in Figure 4c, were quantified (n = 3) and normalized to the signal of the loading control Pgk1 in whole cell extracts (see Figure 4d). Error bars indicate SD; for each pairwise comparison of signals in wildtype (wt) and *Δola1* cells, *p*-values were ≥ 0.05, as determined by a paired two-sided Student's *t*-test.

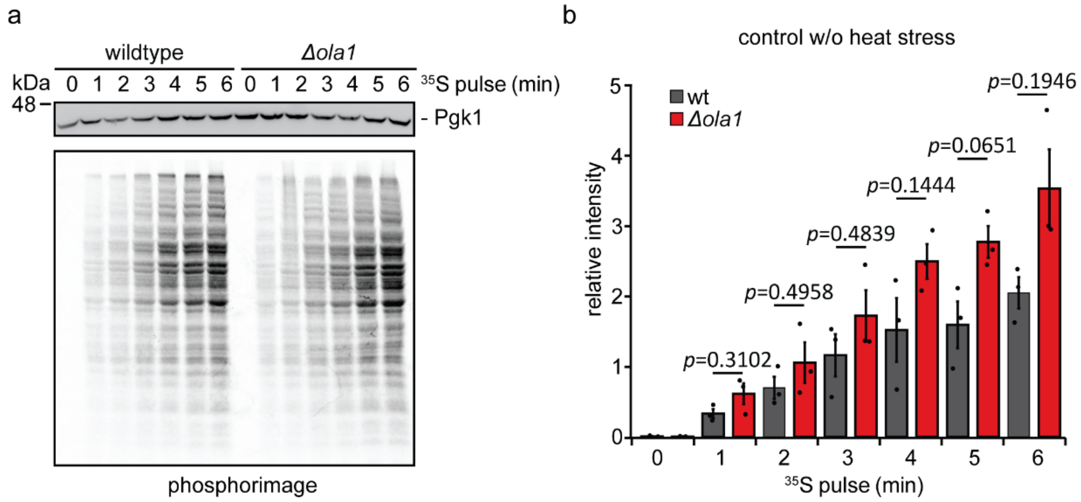

Figure S5

Figure S5. Characterization of translation reinitiation after heat shock relief.

a, b. Wildtype and *Δola1* cells were grown at 30°C. During logarithmic growth, ^{35}S-methionine/-cysteine was added and samples were removed at the indicated timepoints. Incorporation of ^{35}S into newly synthesized proteins was monitored by autoradiography. Aliquots were immunoblotted for Pgk1, which served as a loading control (a). Autoradiographic intensities (n = 3) were quantified and normalized to the loading control Pgk1 (b). Error bars indicate SD; *p*-values were determined using a paired two-sided Student's *t*-test.

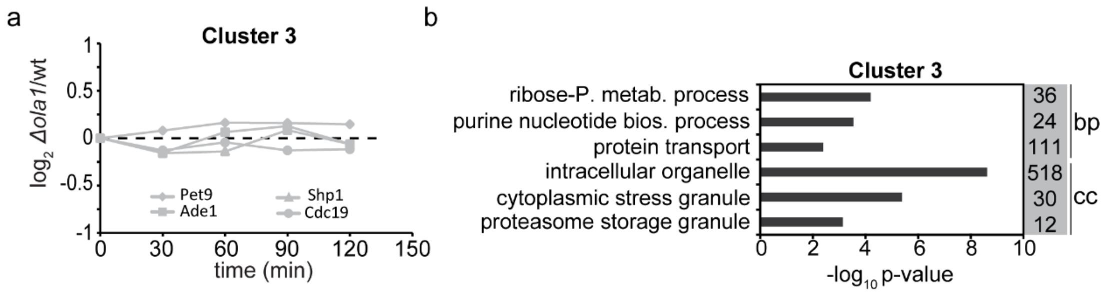

Figure S6

Figure S6. Pulsed SILAC analysis of protein synthesis during recovery from heat shock.

a. Temporal profiles of relative protein synthesis of proteins representative for cluster 3 as determined by hierarchical cluster analysis described in Figure 6.

b. Gene Ontology term overrepresentation analysis of cluster 3 for the domains ‘cellular component’ (cc), ‘molecular function’ (mf), and ‘biological process’ (bp). Listed are selected terms overrepresented and number of proteins assigned to each term that are present in the cluster. ribose-P. metab., ribose phosphate metabolic; bios, biosynthetic.

Supplemental Table Legends

Table S1. Results of the SILAC-based study of effects of heat stress on the proteomes of wildtype and Δ*ola1* cells (a) and GO term enrichment analysis (b). Related to Figure 1 and S1.

a. Whole cell extracts of wildtype (wt) and Δ*ola1* cells treated as indicated in Figure 1a were analyzed by triple native SILAC-based quantitative MS (n = 4). Raw MS data and complete MaxQuant results files are available via ProteomeXchange with the identifier PXD025587. HS, heat stress.
b, c. GO term enrichment analysis of proteins significantly increased/decreased was performed using YeastMine (3). GO terms with a Holm-Bonferroni corrected *p*-value of ≤ 0.05 were considered as overrepresented. bp, biological process; cc, cellular component; mf, molecular function.

Table S2. Results of the SILAC-based study of *OLA1* deletion on the proteome under non-stress conditions. Related to Figure S1.

Whole cell extracts of wildtype (wt) and Δ*ola1* cells were analyzed by SILAC-based quantitative MS (n = 4). Raw MS data and complete MaxQuant results files are available via ProteomeXchange with the identifier PXD025614.

Table S3. Results of native SILAC analysis employed to study effects of heat stress on the detergent-resistant pellet fractions of wildtype and Δ*ola1* cells at 42°C. Related to Figure 2.

a. Detergent-resistant protein aggregates, enriched via differential centrifugation from wildtype (wt) and Δ*ola1* cells, were analyzed by native SILAC-based quantitative MS (n = 3). Raw MS data and complete MaxQuant results files are available via ProteomeXchange with the identifier PXD026323.
b. GO term enrichment analysis of proteins significantly enriched in pellet fractions of heat-shocked wildtype cells was performed using YeastMine (3). GO terms with a Holm-Bonferroni corrected *p*-value of ≤ 0.05 were considered as overrepresented. cc, cellular component.

Table S4. Results of native SILAC analysis employed to study effects of heat stress on the detergent-resistant pellet fractions of wildtype and Δ*ola1* cells at 46°C. Related to Figure 2.

a. Detergent-resistant protein aggregates, enriched via differential centrifugation from wildtype (wt) and Δ*ola1* cells, were analyzed by native SILAC-based quantitative MS (n = 3). Raw MS data and complete MaxQuant results files are available via ProteomeXchange with the identifier PXD025588.
b. GO term enrichment analysis of proteins significantly enriched in pellet fractions of heat-shocked wildtype cells was performed using YeastMine (3). GO terms with a Holm-Bonferroni corrected *p*-value of ≤ 0.05 were considered as overrepresented. bp, biological process; cc, cellular component; mf, molecular function.

Table S5. Results of pulsed SILAC approach employed to study protein synthesis in wildtype and Δ*ola1* cells during recovery from heat shock. Related to Figure 6 and S5.

a. Whole cell extracts of wildtype (wt) and Δ*ola1* cells treated as indicated in Figure 6a were analyzed by pulsed SILAC (n = 3). Raw MS data and complete MaxQuant results files are available via ProteomeXchange with the identifier PXD025673.
b. GO term enrichment analysis of proteins significantly overrepresented in clusters 1-3 as determined by hierarchical clustering was performed using YeastMine (3). GO terms with a Holm-Bonferroni corrected *p*-value of ≤ 0.05 were considered as overrepresented. bp, biological process; cc, cellular component; mf, molecular function.

Table S6. Results of pulsed SILAC analysis employed to study protein synthesis in wildtype and Δ*ola1* cells overexpressing Ola1p during recovery from heat shock. Related to Figure 6g.

Whole cell extracts of wildtype (wt) and Δ*ola1* cells overexpressing Ola1p were treated as indicated in Figure 6a and analyzed by pulsed SILAC (n = 3). Raw MS data and complete MaxQuant results files are available via ProteomeXchange with the identifier PXD025615.

References

1. Dosztanyi, Z., Csizmok, V., Tompa, P., and Simon, I. (2005) IUPred: web server for the prediction of intrinsically unstructured regions of proteins based on estimated energy content. *Bioinformatics* **21**, 3433–3434

2. Prilusky, J., Felder, C. E., Zeev-Ben-Mordehai, T., Rydberg, E. H., Man, O., Beckmann, J. S., Silman, I., and Sussman, J. L. (2005) FoldIndex(C): a simple tool to predict whether a given protein sequence is intrinsically unfolded. *Bioinformatics* **21**, 3435–3438

3. Balakrishnan, R., Park, J., Karra, K., Hitz, B. C., Binkley, G., Hong, E. L., Sullivan, J., Micklem, G., and Cherry, J. M. (2012) YeastMine-An integrated data warehouse for Saccharomyces cerevisiae data as a multipurpose tool-kit. *Database* **2012**, bar062
